# Community engagement in a seaside town: evaluation of Good Grief Weston festival

**DOI:** 10.1177/26323524241274175

**Published:** 2024-09-06

**Authors:** James Robb, Olly Clabburn, Alison Bamford, Fiona Matthews, Karen Lee, Lin Toulcher, Polly Maxwell, Nina Thomas-Bennett, Rachel Hare, Lesel Dawson, Alice Malpass, Lucy E. Selman

**Affiliations:** Palliative and End of Life Care Research Group, University of Bristol, Bristol, UK; Palliative and End of Life Care Research Group, University of Bristol, Bristol, UK; Palliative and End of Life Care Research Group, University of Bristol, Bristol, UK; Super Culture, Weston-super-Mare, UK; Weston-super-Mare Community Network for Health Inequities, Bristol, UK; Weston-super-Mare Community Network for Health Inequities, Bristol, UK; Centre for Death and Society, Bath University, Bath, UK; Weston-super-Mare Community Network for Health Inequities, Bristol, UK; Department of English, University of Bristol, Bristol, UK; Department of English, University of Bristol, Bristol, UK; Palliative and End of Life Care Research Group, University of Bristol, Bristol, UK; Population Health Sciences, Bristol Medical School, University of Bristol, Canynge Hall, 39 Whatley Road, Bristol BS8 2PS, UK

**Keywords:** bereavement, death literacy, evaluation, grief literacy, public engagement

## Abstract

**Background:**

Festivals play an important role in improving death and grief literacy, enabling members of the public to engage with these often-sensitive topics. Good Grief Weston festival was co-designed and delivered with the community in Weston-super-Mare, a coastal town in South-West England with high levels of socioeconomic disadvantage but rich community assets. It was held in person over 8 days in May 2023.

**Objectives:**

To evaluate the reach and impact of Good Grief Weston festival and gather data to inform future festivals.

**Design:**

Mixed methods evaluation (survey and focus groups).

**Methods:**

Online and paper surveys assessing participants’ characteristics and experiences were administrated during and after the festival. Survey participants who indicated their willingness to participate were invited to attend a focus group. Focus groups were recorded, transcribed and analysed using thematic analysis. Data were collected by trained community co-researchers.

**Results:**

Approximately 3000 people attended the festival. Of 204 completed surveys, 64.5% were from women, age range ⩽15 to ⩾75 years; 88.2% identified as White; 14.9% deaf, disabled/with a chronic condition; 18.9% neurodivergent; 9.0% gay, bisexual or queer. Festival participants were entertained (70.9%), inspired (68.5%), felt part of a like-minded community (54.3%), talked to someone new (49.2%), learnt about grief/bereavement (34.3%), shared or expressed experiences (30.3%) and found out about local support (19.7%). 71.3% reported that they felt more confident talking about grief after attending. Median experience rating was 5 (IQR 0; possible range 1 = poor to 5 = excellent). In free-text comments, participants expressed appreciation for the festival and described benefits in attending. Two focus groups were conducted (*n* = 8 participants, all women), lasting c.1.5 h. Focus groups added rich descriptions of the festival’s value, and data to inform the next festival.

**Conclusion:**

Findings suggest festivals of this nature can play a central role in a public health approach.

## Plain language summary

What was the impact of Good Grief Weston festival?

*Why was the study done?* Previous studies have shown that many people feel unsupported by their community after the death of a loved one. We designed a festival to improve knowledge and support for bereaved people. We did this study to assess how the festival affected the people who attended.

*What did the researchers do?* The team included researchers and people and organisations from the local community. Together, we designed and ran a festival on the topic of death, grief and bereavement in Weston-super-Mare, a coastal town with areas of high deprivation. The festival took place over an eight-day period in May 2023 and included creative, interactive and educational events. The team collected feedback on attendees’ experiences of the festival. We did this using online and paper feedback forms, postcards, and focus groups to collect as many opinions as possible.

*What did the researchers find?* We found that a wide range of people attended the festival. Most people who took part in the research rated the festival as ‘excellent’. They told us they learnt about grief and bereavement and found out about local support services. Attendees met new people, shared their experiences, and felt that the festival built community spirit. They also made suggestions to improve any future festivals.

*What do the findings mean?* The findings show that in-person festivals can effectively engage the public on the topic of death, grief and bereavement. They can also help develop connections between people and organisations in communities. These connections and learning opportunities may help communities to support their members through bereavement.

## Introduction

Almost all of us will experience the grief associated with bereavement, yet as a society, we often struggle to talk about it. In a recent survey, 60% of bereaved people in the United Kingdom reported that their community did not help them deal with their grief.^1^ Moreover, experiences of grief and bereavement are unequal. People living in the UK’s most deprived areas are less likely to receive adequate care and support during serious illness or bereavement,^2^ and are more likely to experience social isolation and loneliness – common consequences of serious illness, caregiving and bereavement and major outcome determinants.^3^

### Social deprivation and health in Weston-super-Mare

Weston-super-Mare (WsM) is a coastal town in South-West England. Although the region is relatively affluent, there is substantial wealth disparity; five Lower Layer Super Output Areas in WsM are in the most deprived 5% in England.^4^ WsM has an ageing population, high levels of mental health and addiction problems, and pockets of worsening deprivation.^5^ The population has complex health needs, with 64% of people registered with a Weston Town General Practitioner (GP) reporting having a long-term health condition.^5^ In WsM, worse health is seen in older people, men, ethnic minority groups, lower social classes and people from the most socioeconomically deprived areas.^5^ People who are older, from minoritised ethnic communities and/or living with poverty are known to face specific barriers accessing end-of-life care and bereavement support, including not knowing about available services, discomfort asking for help, discrimination and a lack of appropriate services.^6–8^ The health challenges in WsM are found in many coastal communities across the United Kingdom and have been identified as a priority area by the UK’s Chief Medical Officer.^9^

### Creativity, community assets and their impact

Evidence shows that creative and arts engagement can help reduce inequities and mitigate social isolation, including towards the end of life and bereavement.^10^ In sensitive domains like grief, creative, artistic and cultural initiatives can inform, support and empower people, helping them deal with distress and strengthening resilience.^10^ Festivals, in particular, can be effective ways of engaging and informing the public and improving death and grief ‘literacy’.^11,12^ Engagement and education regarding end-of-life issues are crucial elements of the public health approach to palliative care, which emphasises universal access to palliative care through community ownership and the integration of evidence-based, cost-effective interventions at all levels of healthcare systems.^13^ In WsM there has been increasing community arts investment in recent years. However, resource limitations mean that the resulting initiatives have not yet been fully integrated with existing community assets or the health and social care system. Evaluation of cultural activities has been sporadic with few resources reflecting on lessons learnt.^14^

### Opportunities for change

The transition to Integrated Care Systems (ICSs) in England in 2022 encouraged stronger partnerships between health and social care services and the community. The new system mandates integration between local authorities, the National Health Service (NHS) and other partners, including voluntary, community, faith and social enterprise sectors.^15^ The ICSs’ enhanced partnership reflects the NHS National Palliative and End-of-Life Care Partnership’s Ambitions,^16^ emphasising the need for community partnerships, promoting compassionate communities, improving public awareness and implementing co-design approaches.^17^ In pursuit of these aims, the Arts and Humanities Research Council (AHRC) funded us to establish a cross-sector community network in WsM (The WsM Community Network for Health Inequities) with the aim of working collaboratively to tackle inequities and mitigate social isolation relating to serious illness and bereavement. As part of our programme of work, we conducted and evaluated Good Grief Weston festival.

### Good Grief Weston

Good Grief Weston was an in-person festival about grief and bereavement that took place 1–8 May 2023, with the aim of providing cultural experiences and education on the topics of end of life and grief.^18^ Culture Weston (now Super Culture) led the curation and delivery of the programme, drawing on 15 years’ experience of co-producing inclusive cultural and creative events and activities with WsM communities. The festival programme was co-designed following principles of community engagement, participation and inclusion, working with a range of community organisations, cultural groups, charities and members of the public, and commissioning local artists and creatives working in WsM. The design process and programme were also informed by learning from Good Grief Festival, an online grief festival first held online in 2020.^12^ The programme offered a variety of activities to interest and engage people and promote inclusion (summarised in Table 1; full programme in Supplemental File 1). Several aspects of the programme involved community engagement and activities prior to the festival; for example, c.800 members of the community contributed to the forget-me-nots community artwork project, writing memorial messages on screen-printed, recycled plastic forget-me-nots which were exhibited in a local park (see Supplemental File 2).

**Table 1.** Summary of Good Grief Weston events.

Creative arts	Textile craft with fabrics of personal significance Memorial flower making (forget-me-nots project) Photography project and exhibition Arts, crafts and conversation session Memorial lantern making Bereaved children’s art session Clay memorial tile-making
Performance arts	Youth theatre performances on life, death and memory Requiem of historical musical representations of grief Music and musing with Kathryn Williams Puppetry and poetry
Public engagement, reading and discussion	A ‘reminiscence session’ with historical images Reading groups Talk on the art of memorial tattoos In conversation with Michael Rosen Grief archive exhibition Death Euphemism Bingo Q&A session about death and dying Posters about grief in prominent positions across WsM
Wellbeing and physical expression	Yoga ‘Grief Rave’ on the local High Street Bereaved men’s cookery group Bereavement café Art and nature walk along the coast Synchronised swimming

Events took place in public, private and community spaces across WsM. Venues with limited capacity required pre-booking via an online ticketing system. Events were either free to attend or ‘pay what you can’ with a suggested minimum. The festival cost approximately £25,000 and the evaluation an additional £10,000, excluding academic staff time.

In recognition of the sensitive nature of festival events, ‘listening posts’ were provided, where attendees could seek emotional support from trained volunteers, many of whom were hospice volunteers.

### Aim

The festival aimed to engage the WsM community on the topics of grief and bereavement and raise awareness of existing community assets that help reduce social isolation and inequities relating to end-of-life care and bereavement. The evaluation aimed to assess the festival’s reach and impact to inform future initiatives to engage the public on the subject of grief and bereavement.

## Methods

### Study design

The study design was informed by the Public Health England evaluation framework.^19^ The evaluation methods were co-designed by the research team and community partners. Six local community co-researchers were recruited, trained and collected evaluation data (5 = female; 1 = male). Five were White British and one was mixed race (Korean/Norwegian/British); ages ranged from 31 to 72 years. All co-researchers attended festival events; two were also involved in delivering events (a community workshop and a discussion group). A half-day training session was designed (LES, OC, AM) and delivered (OC, AM) by the academic researchers covering questionnaire administration, obtaining consent, focus group facilitation, responding to participant distress, potential personal impact and the support available.

To offer different ways of providing feedback, we utilised three evaluation methods: a post-event survey (on paper and online); feedback cards; and focus groups.

Findings are reported using the Standards for Reporting Qualitative Research guidelines, as this is a primarily qualitative project.^20^

#### Data collection: survey and feedback postcards

At the end of the festival, an optional online SurveyMonkey Inc.; San Mateo, California, USA; www.surveymonkey.com (Supplemental File 3) was emailed to event registrants. Survey configuration allowed one response per device. Demographic data was collected, including full postcodes in order to calculate Index of Multiple Deprivation Decile.^21^ Free-text boxes enabled respondents to provide feedback or suggest improvements.

During the festival, people who had not pre-registered for events were encouraged to complete the same survey by following a QR link displayed on posters. Paper copies were also available, with completion support provided by community co-researchers, if needed.

Postcard-sized feedback postcards (Supplemental File 4) were also distributed throughout the festival. These provided a participation method for people without access to a digital device (or unable to use one), who preferred not to participate in a written survey or who were under the age of 16. There was no payment for completing surveys or feedback cards.

#### Data collection: focus groups

Survey respondents were asked if they would be interested in participating in a focus group. Willing potential participants provided their contact details and were sent a participant information sheet and consent form via email.

Focus groups were held in person c.4 weeks after the festival and facilitated by four co-researchers (KL, LT, NT, PM) following a topic guide (Supplemental File 5) co-designed by the team based on the literature and prior evaluation.^12,22^ Focus groups were audio-recorded, transcribed verbatim and pseudonymised prior to analysis.

#### Analysis

*Quantitative analysis (survey data)*: Participant characteristics and quantitative data from online and paper surveys and feedback postcards were entered in Excel and summarised using descriptive statistics (JR, AB). Missing responses were excluded from statistical analysis.

*Qualitative analysis*: Focus group transcripts were subject to thematic analysis, incorporating deductive coding (focussed on specific evaluation questions) and inductive thematic analysis (to explore the data).^23^ Familiarisation involved line-by-line reading of each transcript, followed by independent generation of draft coding frameworks (JR, AM, LES) which were then discussed and refined to enhance trustworthiness. Data were coded (JR) according to the final coding framework using NVivo v12 (Lumivero [2017]), retaining the richness of individual participant’s contributions.^24^

Data from paper surveys were entered into Excel (AB) and combined with online survey data prior to analysis. Free-text responses to survey questions (*n* = 130) and feedback postcards (*n* = 50) were coded thematically in NVivo v12 (JR). Seventeen images from feedback cards were categorised by type. Qualitative findings from all data sources were integrated into the narrative results.

## Results

### Quantitative findings

Approximately 3000 people attended the festival with numbers varying between individual events.

#### Participant characteristics

The post-festival survey was completed by 204 individuals (Table 2; median events attended per person = 1; range 1–7). The events attended by most respondents were ‘An evening with Kathryn Williams’ (*n* = 35, 17.2%), ‘In conversation with Michael Rosen (*n* = 31, 15.2%) and ‘Grief at Loves Café festival finale’ (*n* = 25, 12.3%).

**Table 2.** Demographics of survey respondents.

Characteristic	%	*N* = 204
Age group		
0–15	1.0	2
16–19	0.0	0
20–24	2.5	5
25–29	3.0	6
30–34	4.0	8
35–39	9.5	19
40–44	11.6	23
45–49	12.6	25
50–59	24.6	49
60–64	4.5	9
65–69	10.6	21
70–74	9.5	19
75+	6.0	12
Prefer not to say	0.5	1
Sex		
Female	64.5	128
Intersex	0.0	0
Male	33.3	66
Prefer not to say	2.0	4
Gender		
Man	33.0	65
Non-binary	0.5	1
Woman	64.5	127
Not applicable	0.5	1
Prefer not to say	1.0	2
Prefer to self-describe	0.5	1
Ethnicity		
Arab	0.0	0
Asian/Asian British: Bangladeshi	0.0	0
Asian/Asian British: Chinese	0.0	0
Asian/Asian British: Indian	1.0	2
Asian/Asian British: Pakistani	0.0	0
Black/Black British: Black African	1.5	3
Black/Black British: Black Caribbean	0.5	1
Latin American	0.5	1
Mixed Background: Asian and White	0.5	1
Mixed Background: Black African and White	0.0	0
Mixed Background: Black Caribbean and White	0.5	1
White/White British: Gypsy, Roma or Irish traveller	1.0	2
White/White British: White British	81.0	158
White/White British: White Irish	6.2	12
Prefer not to say	1.5	3
Not known	0.0	0
Prefer to self-describe	5.6	11
Disability		
Yes	14.9	29
No	80.5	157
Prefer not to say	4.6	9
Neurodivergent		
Yes	18.9	37
No	78.1	153
Prefer not to say	3.1	6
Sexual orientation		
Bisexual	5.8	11
Gay man	0.5	1
Gay woman/Lesbian	0.0	0
Heterosexual/Straight	81.6	155
Queer	2.6	5
Prefer not to say	6.3	12
Not known	0.5	1
Prefer to self-describe	2.6	5
Currently reside in the United Kingdom		
Yes	97.9	188
No	2.1	4
Occupation		
Clerical and intermediate occupations (such as secretary, personal assistant, call centre agent, clerical worker, nursery nurse)	3.1	6
Full-time education (such as: studying for a degree or apprenticeship)	1.0	2
Long-term unemployed (claimed Jobseeker’s Allowance or earlier unemployment benefit for more than a year)	4.6	9
Modern professional and traditional professional occupations (such as teacher, nurse, physiotherapist, social worker, musician, police officer (sergeant or above), software designer, accountant, solicitor, medical practitioner, scientist, civil/mechanical engineer)	41.3	81
Retired	18.9	37
Routine, semi-routine manual and service occupations (such as postal worker, machine operative, security guard, caretaker, farm worker, catering assistant, sales assistant, HGV driver, cleaner, porter, packer, labourer, waiter/waitress, bar staff)	3.6	7
Senior, middle or junior managers or administrators (such as finance manager, chief executive, large business owner, office manager, retail manager, bank manager, restaurant manager, warehouse manager)	7.1	14
Small business owners who employed less than 25 people (such as corner shop owners, small plumbing companies, retail shop owners, single restaurant or cafe owners, taxi owners, garage owners)	6.6	13
Technical and craft occupations (such as motor mechanic, plumber, printer, electrician, gardener, train driver)	3.1	6
Not applicable (e.g. not currently working and not claiming benefits related to unemployment)	5.1	10
Prefer not to say	4.1	8
Not known	0.5	1
Other	1.0	2
Index of Multiple Deprivation Decile		
1	10.0	13
2	15.4	20
3	13.1	17
4	4.6	6
5	9.2	12
6	10.0	13
7	13.1	17
8	11.5	15
9	7.7	10
10	5.4	7

HGV, heavy goods vehicle.

The majority of respondents identified as women (*n* = 127, 64.5%), heterosexual/straight (*n* = 155, 81.6%), White British or Irish (*n* = 170, 87.2%) and engaged in ‘modern professional and traditional professional occupations’ (*n* = 81, 41.3%) or retired (*n* = 37, 18.9%). The median Index of Multiple Deprivation Decile was 5 (range 1–10). The median age of respondents lay in the 50–59 years category. 8.9% (*n* = 17) identified as lesbian, gay, bisexual or queer; 18.9% (*n* = 37) as neurodivergent and 14.9% (n = 29) as disabled, deaf or living with a long-term health condition.

#### Response to the festival

Survey respondents reported a median rating of 5 for overall festival experience (Interquartile range [IQR] = 0) where 1 = poor and 5 = excellent (*n* = 204). Of the 254 total responses (survey, *n* = 204; feedback cards, *n* = 50), most reported that they were entertained (*n* = 174, 70.9%) and inspired (*n* = 174, 68.5%) by the festival. One hundred thirty-eight respondents (54.3%) felt part of a like-minded community and 125 (49.2%) spoke to someone new. Others felt they had learnt about grief and bereavement (*n* = 87, 34.3%), were able to share or express their experiences (*n* = 77, 30.3%), or found out about local bereavement support or community groups (*n* = 50, 19.7%).

#### Attitudes towards communicating with bereaved people

Participants were asked about their attitudes towards communicating with bereaved people. 71.2% (*n* = 131) said: ‘I would know what to do if someone who was recently bereaved told me they were having trouble’, and 71.2% (*n* = 140) reported: ‘I would know what kind of help or support to offer someone who was bereaved’. Conversely, 70 people (37.8%) agreed with the statement: ‘I would be scared of “saying the wrong thing” to someone who was recently bereaved’ and 28 (14.8%) with the statement: ‘I would avoid talking to someone who was recently bereaved about their bereavement because I wouldn’t know how to help’. 71.3% (*n* = 139) of participants reported that through attending the festival they felt more confident talking about grief.

#### Accessing bereavement support

24.3% (*n* = 96) of survey respondents stated that they would primarily seek bereavement support for themselves or someone else from a GP or doctor, and 18.0% (*n* = 71) from local hospice. 16.7% (*n* = 66) said that they would not seek professional support, relying instead on family and friends. Through attending the festival, 48.2% (*n* = 92) said that they had learnt about local services and support for people living with serious illness or bereavement.

### Qualitative findings

Of the 204 survey respondents, 64 (31.4%) expressed interest in focus group participation. Eight White British/Irish women aged 25–39 (*n* = 1), 40–59 (*n* = 3) and 60+ (*n* = 4) participated in a focus group which lasted 64–81 min.

A total of 130 survey respondents (63.7%), provided free-text comments about their experience and 99 (48.5%) offered suggestions to improve future festivals.

Forty (80%) postcard respondents wrote free-text comments and 17 (34%) drew pictures. Pictures included images of smiley faces (*n* = 8), love hearts (*n* = 3), nature (*n* = 3), figures embracing (*n* = 1) and abstract doodles (*n* = 1) (select images in Supplemental File 6).

#### Awareness of the festival

Many focus group participants were motivated to attend the in-person festival following previous positive experiences of the online iteration.^12^ Some saw the festival as an opportunity for ‘healing’ (P3FG1) and to engage with unresolved grief. Participants were mostly from the WsM area and keen to support a local event:

I actually couldn’t believe it when I saw that the first live Good Grief events were going to be in [WsM] of all places! So I really, I wanted to attend the ones that I wanted to go to because of that and also just to support the fact that it was being done in Weston. (P7FG2)

Some participants learnt about the festival through social media (Facebook and Instagram) or emailing lists (from prior Good Grief participation). People who didn’t use social media relied on word of mouth or physical advertisements (e.g. flyers and posters in local cafes and hospices), though these went unnoticed by some. Similarly, advertising on local radio and newspapers went unrecognised by some participants who reflected that these platforms, and local TV, could widen their reach. Some suggested more decoration might help advertise the festival:

I thought the posters were really, really good. It’s just that I didn’t see them dotted around. I didn’t know it was advertised so that’s why I was thinking if it happens again next year to [. . .] just brighten up the town with things, not just posters, just some forget-me-nots, massive sunflowers that are made out of art that are in the high street just dotted around [. . .] to say that there is a festival happening here in Weston. (P2FG1)

‘Publicity not sufficient’ (respondent 71) was a common theme for survey respondents, as was the suggestion to ‘advertise more widely, “prime the pump” throughout the year, [and] link to other events/mourning’ (respondent 66). Others felt the problem was more nuanced: ‘I know there was loads of advertising but somehow it only reached some of the population – maybe those already in the loop – how to extend?’ (respondent 98).

#### Perceptions of programme and format of events

Focus group participants felt that the programme was broad with ‘something for everybody’ (P5FG2) and similarly catered to their own interests. People were attracted to activities they knew would be enjoyable or provide a new experience:

I was really excited when I saw [. . .] the range of activities that were on. I think the range of the programme it was almost all the things I love doing, right down to I’ve always wanted to be a synchronised swimmer, and yoga and lots of things. (P3FG1)

Some were concerned that the programme could be overwhelming, but acknowledged that the breadth enabled attendees to focus on events of specific interest:

I thought blimey that’s a pretty full-on programme. That could be really overwhelming for some people but then I thought not everybody’s gonna go to all of it and they’ll be drawn to whatever they’re drawn to for whatever reason, but I was a little bit nervous thinking how much shall I go to, am I gonna get overwhelmed? (P8FG2)

Pre-booking online was required for some events. This could present a barrier through digital exclusion or logistical challenge, especially for recently bereaved people: ‘It puts me off a bit’ (P5FG2). In contrast, events where participants could ‘turn up’ were attractive as they removed these logistical barriers:

If you’re really having trouble grieving you wouldn’t be able to get your head round booking all these things. (P8FG2)

But then I also think you can dip your toe in [. . .] I think it’s very drop-in-able, isn’t it? And I think that’s what you need when you’re feeling, oh, a bit anxious. (P3FG1)

Clashes with work schedules were commonly cited barriers. Others noted that timing the festival over a bank holiday weekend likely increased attendance. A small number of events were especially popular and were fully booked, leading to disappointment. As such, some survey responses called for greater capacity at events with high-profile guests.

Focus group participants who were professionally engaged with grief and bereavement (e.g. counsellors and art therapists) reflected that prior to the festival they were apprehensive that certain events might overwhelm people. Another participant said that understanding her own grief was self-protective, but felt that elements of the festival might be triggering for others:

I’m a therapist [. . .] I looked at [the programme] and thought God, I hope there’s gonna be a load of counselling people that can support people ‘cause this could really trigger people big time. (P8FG2)

I was very much aware unfortunately that week coincides with the anniversary of my late husband’s death and while I’m okay with it I know for those few days I’m a little bit needing to be withdrawn. I know how to manage it so that’s why I was very careful and picked what I felt able to go to ‘cause I knew I was gonna go to everything on my own so that, wasn’t an issue for me but I can well understand how it could so easily trigger somebody maybe who hadn’t had time to sit down and think about it. (P4FG2)

#### Experience of a grief festival located in WsM

Some participants were surprised that the festival was held in WsM as they perceived it as a neglected place. Hope was felt that such events could be revitalising and draw people back to the area:

[. . .] things like this just never happen in Weston. It’s such a forgotten about place so, yeah, I think it’s really important for things like this to happen here because other places get prioritised, like [nearby city]. (P1FG1)

Such a great idea for Weston and community, bringing life back into this town I loved as a child for our next generation. (Survey respondent 164)

It was important for participants that the event was run in collaboration with the community and not dominated by external actors. They valued the inclusion of local people in the events:

It didn’t feel like [name of University] was swamping into Weston. It felt like it used the community centres and the community hubs to grow the festival out of it. (P3FG1)

Lots of the people that I know, lots of the other artists and hosts, most of them were Weston-based. Obviously, some of the big people, like Michael Rosen, they came from other places, but lots of people were home grown, from here. (Focus Group Facilitator 2)

#### Responses to events attended

Participants attended a broad range of events, said the festival was ‘well-organised’ (P5FG2) and run ‘professionally’ (P7FG2). They described events as ‘welcoming’, ‘rich’, ‘a brilliant concept’, ‘wonderful, friendly, and a comfortable experience’ (survey respondents).

Focus group participants described how events promoted creative expression and processing of grief. Some described feelings of liberation, and one person described therapeutic benefit of hosting an event:

[The festival was] creative, facilitating some of the release of my, of people’s emotional layers or denial of grief. (P8FG2)

I thought it was deep and free. . . Freeing. (P7FG2)

Enlightening and I’d say yeah, creative. (P6FG2)

I really liked the Forget-Me-Not Project and being able to host an event as well [. . .] I felt it was really sort of healing, you know, being with different people and the conversations we had. It was not sombre but [. . .] it felt really special. (P3FG1)

Some survey respondents reflected that in UK society grief and bereavement are taboo or stigmatised. The opportunity to challenge perceptions through the festival was welcomed:

It’s wonderful to have events that encourage you to think about your losses and that help you feel like it’s ok to think about them. It can be very difficult to discuss grief in our society, which is weird because everyone has experienced it. There is a tendency to expect people to move on within an imaginary time-frame, or to at least keep it to themselves if they haven’t. It’s beautiful to be able to shed a tear with someone without feeling silly or ashamed, sharing a feeling, which is very personal, but also very shared and familiar. I hope this happens every year. (Survey respondent 200)

Another respondent stated that the event was ‘like a political social movement [. . .] bringing wisdom back to the community, away from corporations’ (PC47).

Accessible forms of artmaking such as clay sculpture and tile painting provided opportunities to express emotion and memorialise loved ones. While some participants were accustomed to making art as an emotional outlet, others were not regularly creative due to negative experiences at school. These people found creativity empowering:

I’ve not really expressed my grief in that way [. . .] I really didn’t know what I was gonna do but it really flowed really well and I was just so moved and it felt really special and it really did help me grieve ‘cause I felt like I’d done something that’s gonna be outside that I can go and visit that, and my dad was and my sister was looking down on me and I was like, you know, I did you proud ‘cause you’ve got something. (P8FG2)

Several participants said that events made them feel unexpectedly emotional, even when they thought they were prepared. One person reflected that this might be due to the proximity of other bereaved people. Others expressed that feelings of loss were unexpectedly triggered:

I think grief is so hard [. . .] I went to one of the sessions, it was one of the art sessions, [. . .] and I just burst out crying. I couldn’t cope with it. [. . .] I was just ‘oh’, I was a bit surprised about that and I had to sort of get out of that session. I think it can catch you at different times, even though you think you’re prepared and ‘oh, that’s what I’m going to’, it was too intense almost being with other people who’d had losses. I sort of couldn’t cope with it. (P3FG1)

I went to find mine [decorated memorial flower]. I could not find mine and I walked up and down and it suddenly became quite important actually. I mean, my husband [would] say ‘oh, what are you fussing about?’, but actually I kind of walked away with a little bit of a feeling, that sense of loss. (P6FG2)

Participants generally reported that events were safe spaces, where ‘you are not going to be judged’ (P1FG1) and there was mutual understanding and support between attendees:

[. . .] you could go to the events on your own is what I was saying. It felt quite safe. [. . .] It was really safe and inclusive and I was really trying to get somebody to come to the synchronised swimming and then the people were ‘no, no, we’ll watch, we’ll watch’ and then it was just like ‘well, no, I’m going to do it’ and again the group from [nearby city] was so lovely and so inclusive. It was just, you couldn’t stop smiling. It was just really lovely. Really, really lovely. (P3FG1)

‘Listening people’ were provided by the festival to offer emotional support during events, and participants valued them:

If you felt upset during the film there were people who you could go and talk to and stuff, so I think it’s having people like that and people knowing that there’s a safe space and you can leave if you want to. I think it’s a really good part of it, definitely. (P1FG1)

One participant felt this support could have been better promoted. Two participants suggested that it might be helpful to have more emotional support from trained and supervised staff available at the next festival; interestingly, both the participants expressing this view were therapists.

#### Finding balance and generating connections

Participants acknowledged the importance of a festival explicitly dealing with grief and bereavement but also valued events that were joyous and celebratory:

You had to have a little bit of light with the dark. I went oh, [synchronised swimming] is a bit weird, why have they got that? And then I realised you couldn’t go to all of those things being so heavy without the light things as well. (P8FG2)

One participant expressed a desire for more events which celebrate life, and suggested that framing the festival this way might increase attendance:

Shame [it wasn’t called] ‘Love, Loss and Celebrating Life’ because I think some people were, not put off, but thought it would be too sad. (P2FG1)

Some participants felt that it might be beneficial to offer opportunities to debrief with other attendees after emotionally challenging events.

Participants described the festival as facilitating connections and conversations between attendees, for example through sharing memories of loved ones, which in turn promoted a sense of belonging:

It’s been amazing to be part of this and to book other events during this week. It’s nice to feel a sense of belonging with others who have also experienced grief. (Survey respondent 133)

One focus group participant working in end-of-life care was surprised to learn about organisations they weren’t aware of that would be beneficial to hospice service users.

#### Inclusivity and diversity

Participants felt that the festival was inclusive but identified ways to enhance future events, including initiatives which encourage more men and young people to attend. Focus group participants suggested high-profile male speakers, sport activities and ‘Men’s Sheds’ events could further engage men.^25^ The median overall experience rating for men (*n* = 65, 33%) was 5 (excellent), and most free-text responses were very positive. The focus group participants reported that male relatives had specific challenges expressing grief, and noted festival events that might engage them:

It’s funny actually ‘cause my dad had some terrible losses in his life [. . .] but he had cats and it was when the cats died that was when he was able to grieve because somehow he couldn’t talk or cry about the other losses. (P6FG2)

He loves gardening. That’s been the way that he’s processed his grief. You know, the garden’s become a real focus, and you had an allotment type session, didn’t you, and there was that walk as well, [. . .] I think whoever organised it had really thought about different things. (P3FG1)

Focus group participants reflected that the festival was predominantly attended by women. Some felt that the gender balance was ‘gonna be the case’ (P8FG2). One participant stated that her male partner was reluctant to attend events where he anticipated attendees to be mostly women:

He was like ‘oh, I’ll go to the yoga ones’. The other ones he thought, as a man’s point of view maybe, for him it was like ‘oh, I don’t think I’ll go to the arts one [. . .] it will all be women [. . .] I don’t think I’ll go’. So, maybe just like a men’s circle. (P2FG1)

Some focus group participants felt that there could be more provision for children and young adults, suggesting including music, dance and gaming in future festivals.

One survey respondent requested more representation of faith groups and another referred to folk traditions. There was no explicit discussion of ethnic and cultural diversity in focus group conversations.

The median experience rating for neurodivergent people (*n* = 37, 18.9%) was 5 (excellent). Most free-text survey comments were positive. Respondents had mixed experiences of event accessibility for disabled people, likely due to of event type and location. Several focus group participants remarked on good accessibility of events they attended, and the median experience rating was 5 (excellent) for survey respondents who were D/deaf and/or D/disabled (*n* = 29, 14.9%). Two survey respondents (with additional mobility needs) stated that they were unable to attend desired events due to lack of public transport, level access or space limitation at venues. Ensuring accessibility of event locations with more dedicated spaces to rest were suggested:

There needs to be more info about venues and what buses may help. If you’re going to have a speaker as popular and helpful as Michael Rosen use a much larger hall or book for two sessions. I would have gone to other [events] if I could have got there as [I have] bad arthritis and little sight. (Survey respondent 5)

Another participant reported that alcohol consumption at a minority of events might present a barrier to those with a history of substance use:

My friend came to the door in the afternoon and she’s alcoholic and she said ‘I couldn’t walk in ‘cause the smell of alcohol was putting me off’ and I did think this is really bad that people grieving who are alcoholics can’t now enter the building. I didn’t notice the smell of strong alcohol but I did notice a lot of people were drinking and I did think ‘oh, that’s gonna put a lot of people off from coming in’. (P8FG2)

Feedback from survey respondents from the LGBTQ+ community (*n* = 17, 8.8%) were almost all positive, but one participant stated:

Great concept. Some of the groups were not LGBTQI+ safe. One group, in particular, was felt to have been overly ‘clicky’ and therefore felt exclusive. (Survey respondent 187)

#### Space as a mediator of participation

Participants appreciated that the festival used community assets such as community hubs, creating opportunities to explore parts of town not previously visited. Physical space was felt to be important in creating the right atmosphere for participation. Due to inclement weather, a planned event finale in a park had to be moved to a local café, which did not suit all participants. Some people reflected that events in confined spaces felt overwhelming:

I couldn’t cope with going out and facing people. I did get overwhelmed going into [café hosting event] on that Sunday ‘cause there was so many things and stalls and groups of people. (P8FG2)

Open spaces were perceived as ‘safer’, allowing for peripheral participation. There was a desire for future events in local parks. Some participants commented that a ‘festival hub’ could act as a permanent fixture throughout the week, and be used after events to help people ‘decompress’:

On one of the days someone might just think ‘well, I don’t really feel like going to any of these but I do feel like being around someone at the moment so I’ll just sit in the park and chat to someone, or not even chat to anyone or just sit with a blanket, but there’s something going on in the park. (P2FG1)

Yeah. And actually, when I’m sat in the park I might then want to get involved. (P3FG1)

If you knew there was something happening at the park, like food and music and stuff people might feel they can kind of gravitate towards there. (P1FG1)

Most participants valued the in-person format because it facilitated greater participation and interaction. One participant highlighted the benefit of online events for those who were physically or emotionally unable to attend in-person. Others expressed appreciation for existing online Good Grief resources and previous online events, especially during lockdown^26^:

[Good Grief Weston] was wonderful but I actually got a lot in lockdown from some of the online festival. (P6FG2)

## Discussion

Approximately 3000 people attended Good Grief Weston. Overall, evaluation findings suggest that the festival successfully inspired, engaged and educated people on the topics of grief and bereavement, and benefitted this often-neglected coastal town by bolstering community pride and bringing people together.

The respondents were mainly women (64.5%), likely reflecting lower engagement by men with both the festival and the post-event survey. Similar patterns have been reported elsewhere^12,27–29^ and may reflect findings that during bereavement men tend to prioritise independence and express symptoms and emotions less than women.^30^ However, the gender balance is more equal than at the first online Good Grief Festival in 2020, where 90% of respondents to the post-festival survey were women.^12^ The focus group participants highlighted some of the ways in which their male relatives struggled to express emotions related to grief, but also noted that they were engaged by festival events. The combination of these findings indicates some success in efforts to increase male participation with Good Grief initiatives through incorporating specific events designed to engage men.

Good Grief Weston attracted people from diverse socioeconomic backgrounds (median Index of Multiple Deprivation (IMD) = 5; range 1–10), with 25% of attendees from IMD deciles 1 and 2 (most deprived). A diverse socioeconomic group was also represented in focus groups (median IMD = 6; range 2–10). Discourse around death and dying is often dominated by middle-class norms,^31^ and little is known about the end-of-life priorities of people from lower socioeconomic groups.^7^ Festival attendance by people from more deprived neighbourhoods is likely to reflect the co-production approach and outreach, for example via offering participation in the forget-me-nots project at community hubs in some of WsM’s poorest neighbourhoods. Hansford et al.^7^ caution that public health palliative care initiatives must be deeply rooted in the communities in which they operate, to avoid inadvertently perpetuating inequality.

The proportion of survey respondents from minoritised ethnic groups, however, was lower at Good Grief Weston (5.6%) compared with the 2020 online festival (9%).^12^ Future local festivals should aim to improve inclusion and diversity, for example by co-producing events and activities with diverse communities, linking with faith groups and ensuring representation among facilitators.^12,32^ Comments from LGBTQI+ participants, who experience additional stressors in bereavement including homophobia and transphobia, failure to recognise queer relationships and additional financial and legal burdens,^33^ were overwhelmingly positive, however, one participant felt one event space was not inclusive, which can inform future events. Experiences of people who were deaf, disabled or living with a long-term health condition, or were neurodivergent, were generally positive. Previous research has indicated that people with learning disabilities may require additional bereavement support due to an increased risk of secondary loss and differences in expression and communication.^34,35^

There are relatively few public engagement initiatives dealing explicitly with death and bereavement,^36–38^ and even fewer with published evaluations; those identified are all UK-based.^12,28,29,39^ The findings from this festival evaluation are broadly comparable to existing evidence: attendees appreciate the opportunity to discuss grief; find it helpful to share their experience; and think it is important to discuss death, dying and loss. The co-production design enabled local people to meaningfully contribute to the festival, and engendered a sense of ownership which is absent from events which are less community-led.^29^ In addition, the decentralisation of activity provision may have helped reduce the emotional labour demanded of facilitators described elsewhere.^28^ The evaluation of ‘This Grief Thing’ also reports the generation of connections between participants, local organisations and creative arts groups, but notes that measuring the less concrete ‘ripples’, such as intimate person-to-person interactions, is complex.^28^ Our finding that festivals could have a regenerating effect on the local area is novel among bereavement related events, although the potential for festivals to contribute to the local economy and strengthen cultural assets is well-documented.^40^

The reported benefits of the first online Good Grief Festival included widening access to information and support, the ability to engage from one’s own home and being able to dip in and out of sessions.^12^ This evaluation of Good Grief Weston has shown that in-person events are effective at fostering environments in which participants can mutually support each other and connect with others. Attendees valued the informal support facilitated by the workshops and events, evidenced by the high proportion of people who talked to someone new, felt part of a like-minded community and shared or expressed their experiences. Some focus group participants who worked in bereavement services were concerned that a grief festival could cause distress and called for additional ‘listening posts’. However, while there was qualitative evidence of festival attendees expressing emotion, we received no reports of harm, and participants described seeking and receiving emotional support from fellow workshop attendees. Festival events may therefore be a useful way of facilitating effective community and peer-to-peer support.^41^ Strengthening informal support networks can bolster communities’ response to grief and bereavement, mitigating the professionalisation of grief and potentially allowing professional services to be reserved for those who need them most.^42^

The results of this study have several implications. The respective evaluations of Good Grief Weston (in-person) and Good Grief Festival (online) indicate that each format successfully achieved the primary aim of engaging the public on the topic of grief, bereavement and death. However, this evaluation has illustrated how each configuration has its own unique set of benefits and limitations which can be used to inform future festivals. Our findings further suggest that festivals can play an important role in public health approaches to bereavement support, connecting community members while raising awareness and increasing confidence, and therefore may contribute towards meeting the recommendations of the UK Commission on Bereavement.^43^ Our experience of evaluating this festival, particularly the training and support of community co-researchers to collect data, can also contribute to existing methods for assessing such interventions.^22^

The strengths of this study include using a mixed methods design to enable both quantitative and qualitative insight into attendee experiences.^44^ The festival and the evaluation were co-designed with community partners, ensuring that provision reflected the needs of the local population and that the evaluation methods were appropriate.^45^ The evaluation was enhanced through collaboration with community co-researchers who are embedded in and trusted members of the local community, and who were trained and paid. The study benefitted from their local knowledge and helped to build local capacity in research methods.^46^ Data were collected via various means to facilitate participation from a broad range of attendees (e.g. people under the age of 16 or who prefer drawing to writing).

There were limitations to this study. Only a relatively small proportion of the estimated number of people who attended the festival participated in the evaluation. The evaluation included only those who attended festival events; we did not collect data from people who were physically or emotionally unable to attend, or who only participated in community activities (e.g. the forget-me-nots project) prior to the festival. Moreover, attendees who felt strongly positively or negatively might have been more motivated to participate in the survey or a focus group. Two of the co-researchers involved in the evaluation were also involved in delivering festival events; participants were not told this information, however, if they were aware of it this may have biased their responses. Whilst the online survey was configured to permit only one submission per device it was technically possible for respondents to submit more than one paper survey. The lack of diversity in focus group participants is a limitation: their experiences do not reflect the range of people who attended the festival, and while they provided suggestions for increasing engagement with people from minoritised groups, men and children, they did so from the perspective of White women. Future festival design should continue to attend to co-production principles to ensure that events reflect the desires and needs of diverse local communities. Surveys, feedback cards and focus groups were available only in English, excluding those who do not speak or write in English from giving feedback.

The findings of this study highlight several priorities for future research. A recent review highlights the potential for creative health approaches to improve health outcomes and the role of culture and creativity in improving the places people live and strengthening communities.^47^ Further research is required to explore the role of grief festivals in system-based approaches that aim to reduce inequities towards the end of life and bereavement, and/or mitigate associated social isolation and loneliness, and the cost-effectiveness of festivals compared with other methods. Of note, several festival events were very low cost (e.g. creative community workshops, reading groups, yoga), while others built on existing community initiatives and activities (e.g. the youth theatre, bereavement café), or set up new initiatives which have had a lasting legacy (e.g. synchronised swimming group, memorial tile-making). To inform future work, the experiences of specific groups, including people from diverse ethnic backgrounds, younger people and men, warrant further research to identify barriers to – and facilitators of – engagement.

The replicability of outcomes from this festival must be tested in other UK coastal communities and beyond. The next phase of our work, also funded by the AHRC, involves collaboration with two other coastal towns in the United Kingdom to further investigate the role of cross-sector partnership, community assets and public festivals and events in mental health and wellbeing.^48^ Further research is required to assess whether such festivals can have lasting impacts on the attitudes of local people and whether the benefits lead to improved experiences of grief and bereavement and a reduction in inequities in this field. Methods for evaluating these complex interactions will need to be developed.

## Conclusion

Good Grief Weston successfully engaged a large number of diverse people on the subject of grief and bereavement. Attendees enjoyed the experience and became more confident discussing end-of-life issues. The festival facilitated mutual support between community members, with attendees reporting that they felt part of a like-minded community and citing numerous benefits for the host coastal town. Findings support the idea that festivals can engage communities and facilitate changes in attitudes towards important, yet sometimes challenging, subjects such as grief and bereavement. Festivals might also help build the capacity of coastal communities to respond to their unique health challenges by facilitating connections and awareness of services. Further research is warranted to explore the long-term impact of festivals of this nature on community health and wellbeing.

## Supplemental Material

sj-docx-1-pcr-10.1177_26323524241274175 – Supplemental material for Community engagement in a seaside town: evaluation of Good Grief Weston festival Supplemental material, sj-docx-1-pcr-10.1177_26323524241274175 for Community engagement in a seaside town: evaluation of Good Grief Weston festival by James Robb, Olly Clabburn, Alison Bamford, Fiona Matthews, Karen Lee, Lin Toulcher, Polly Maxwell, Nina Thomas-Bennett, Rachel Hare, Lesel Dawson, Alice Malpass and Lucy E. Selman in Palliative Care and Social Practice

sj-docx-3-pcr-10.1177_26323524241274175 – Supplemental material for Community engagement in a seaside town: evaluation of Good Grief Weston festival Supplemental material, sj-docx-3-pcr-10.1177_26323524241274175 for Community engagement in a seaside town: evaluation of Good Grief Weston festival by James Robb, Olly Clabburn, Alison Bamford, Fiona Matthews, Karen Lee, Lin Toulcher, Polly Maxwell, Nina Thomas-Bennett, Rachel Hare, Lesel Dawson, Alice Malpass and Lucy E. Selman in Palliative Care and Social Practice

sj-docx-4-pcr-10.1177_26323524241274175 – Supplemental material for Community engagement in a seaside town: evaluation of Good Grief Weston festival Supplemental material, sj-docx-4-pcr-10.1177_26323524241274175 for Community engagement in a seaside town: evaluation of Good Grief Weston festival by James Robb, Olly Clabburn, Alison Bamford, Fiona Matthews, Karen Lee, Lin Toulcher, Polly Maxwell, Nina Thomas-Bennett, Rachel Hare, Lesel Dawson, Alice Malpass and Lucy E. Selman in Palliative Care and Social Practice

sj-docx-5-pcr-10.1177_26323524241274175 – Supplemental material for Community engagement in a seaside town: evaluation of Good Grief Weston festival Supplemental material, sj-docx-5-pcr-10.1177_26323524241274175 for Community engagement in a seaside town: evaluation of Good Grief Weston festival by James Robb, Olly Clabburn, Alison Bamford, Fiona Matthews, Karen Lee, Lin Toulcher, Polly Maxwell, Nina Thomas-Bennett, Rachel Hare, Lesel Dawson, Alice Malpass and Lucy E. Selman in Palliative Care and Social Practice

sj-docx-6-pcr-10.1177_26323524241274175 – Supplemental material for Community engagement in a seaside town: evaluation of Good Grief Weston festival Supplemental material, sj-docx-6-pcr-10.1177_26323524241274175 for Community engagement in a seaside town: evaluation of Good Grief Weston festival by James Robb, Olly Clabburn, Alison Bamford, Fiona Matthews, Karen Lee, Lin Toulcher, Polly Maxwell, Nina Thomas-Bennett, Rachel Hare, Lesel Dawson, Alice Malpass and Lucy E. Selman in Palliative Care and Social Practice

sj-jpg-2-pcr-10.1177_26323524241274175 – Supplemental material for Community engagement in a seaside town: evaluation of Good Grief Weston festival Supplemental material, sj-jpg-2-pcr-10.1177_26323524241274175 for Community engagement in a seaside town: evaluation of Good Grief Weston festival by James Robb, Olly Clabburn, Alison Bamford, Fiona Matthews, Karen Lee, Lin Toulcher, Polly Maxwell, Nina Thomas-Bennett, Rachel Hare, Lesel Dawson, Alice Malpass and Lucy E. Selman in Palliative Care and Social Practice

## Declarations

**Ethics approval and consent to participate:** This research was conducted according to the World Medical Association Declaration of Helsinki. Ethical approval to conduct the evaluation was granted by the University of Bristol Faculty of Health Sciences Research Ethics Committee (Ref: 2023-14220-15977) on 24 April 2023. Written informed consent to participate was obtained from participants.

**Consent for publication:** Informed consent to publish was obtained from participants.

**Availability of data and materials:** Study data will be deposited at the University of Bristol Research Data Repository (data.bris.ac.uk/data) from 1 January 2025, stating how data can be accessed by bona fide researchers. After this date, requests for access should be directed to the Research Data team at Bristol, who will assess requests before granting access. No authentic request for access will be refused and re-users will not be charged for any part of this process.
