# Supplementary figures and images for: Community engagement in a seaside town: evaluation of Good Grief Weston festival
Source: Palliat Care Soc Pract. 2024 Sep 6;18:26323524241274175. doi: 10.1177/26323524241274175 (PMC11378171; doi:10.1177/26323524241274175)

# Supplementary file 1 – Good Grief Weston programme of events
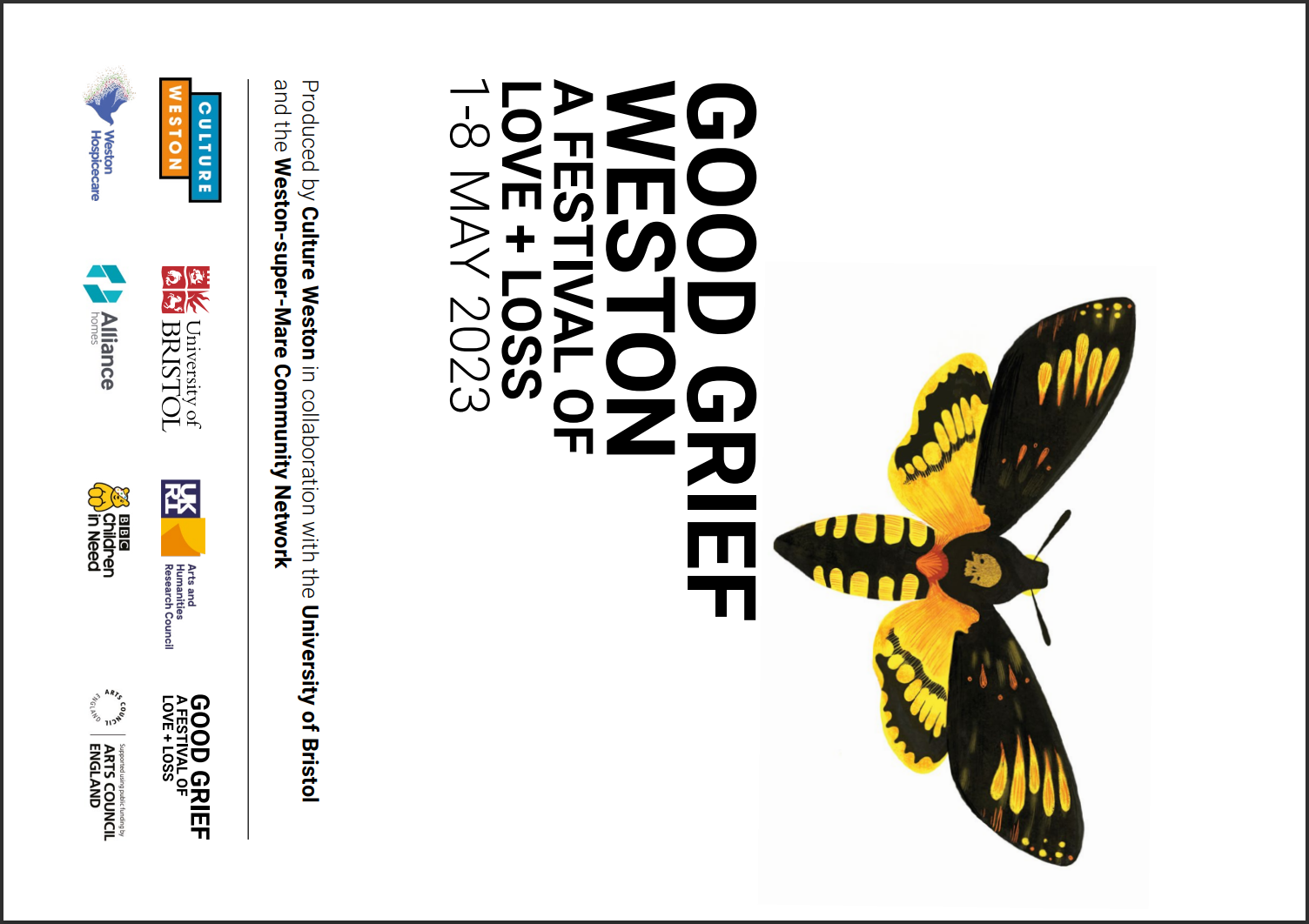


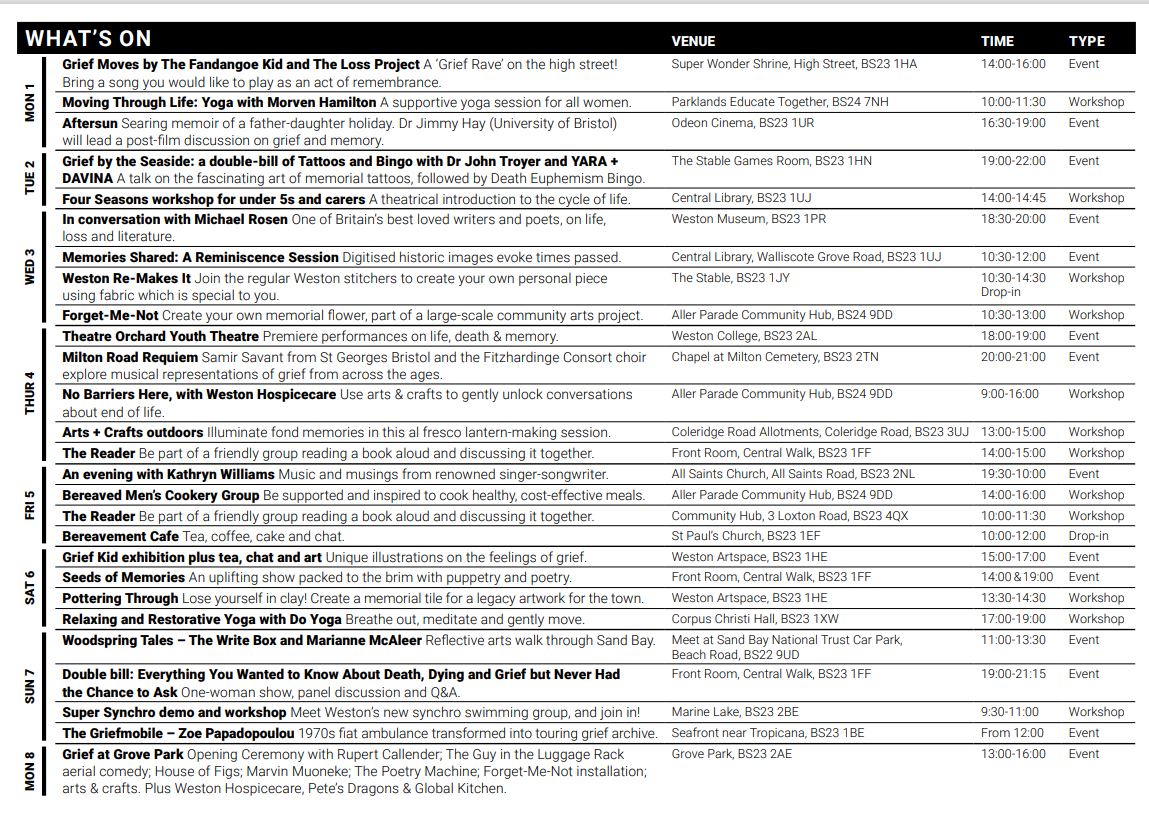


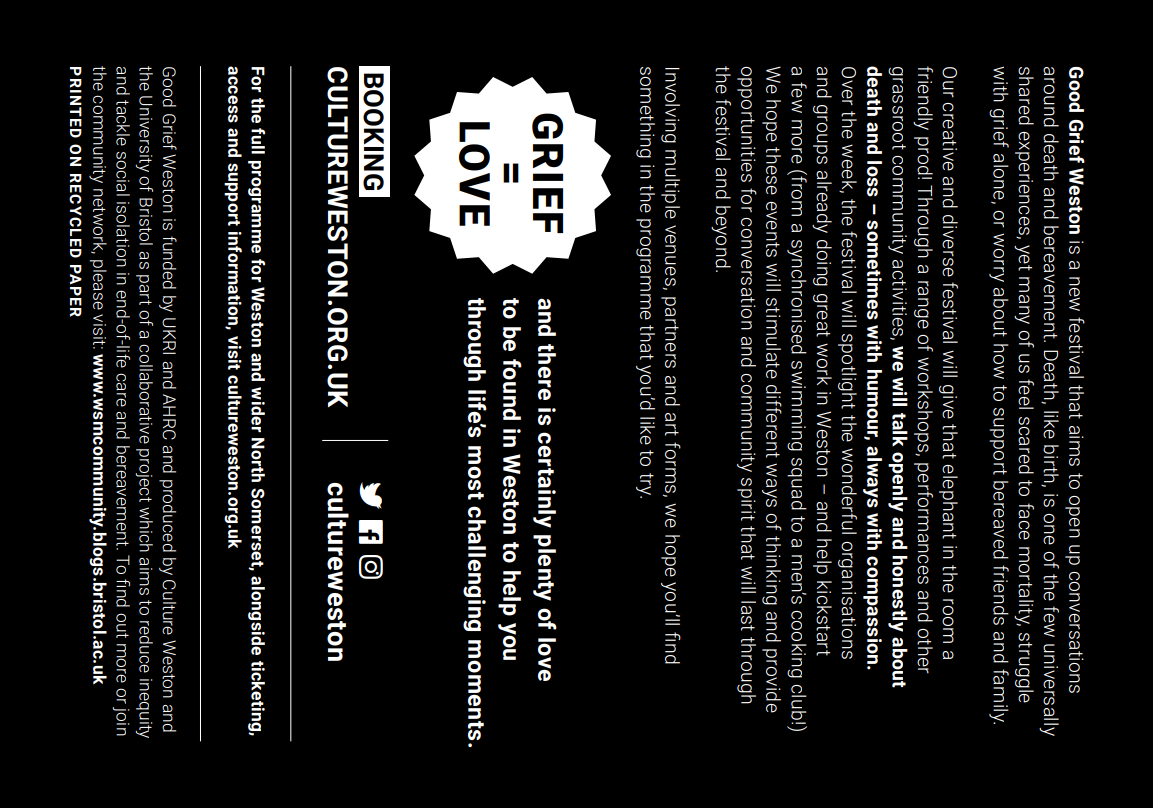

Supplement: sj-docx-1-pcr-10.1177_26323524241274175 – Supplemental material for Community engagement in a seaside town: evaluation of Good Grief Weston festival [file sj-docx-1-pcr-10.1177_26323524241274175.docx]

Supplementary file 4 – Feedback card
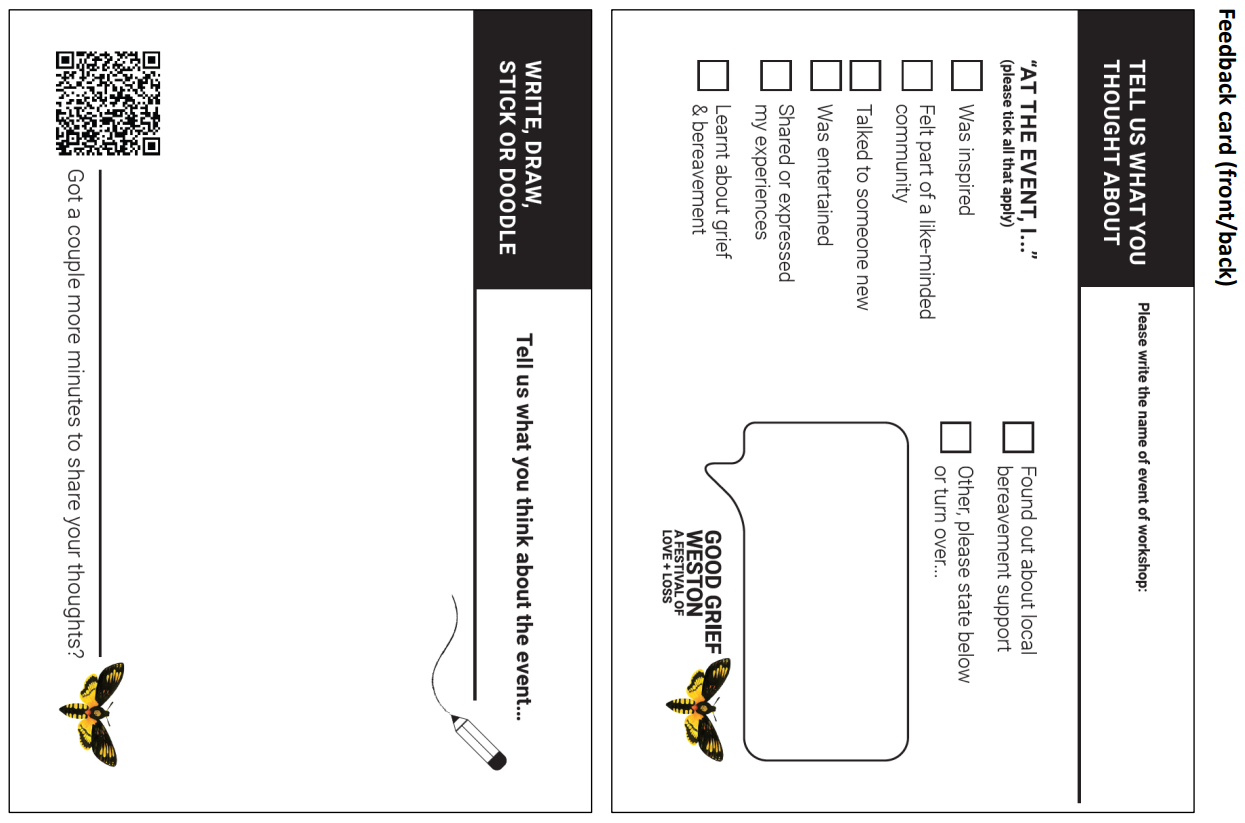

Supplement: sj-docx-4-pcr-10.1177_26323524241274175 – Supplemental material for Community engagement in a seaside town: evaluation of Good Grief Weston festival [file sj-docx-4-pcr-10.1177_26323524241274175.docx]

Supplementary file 6: Selection of images from postcard feedback


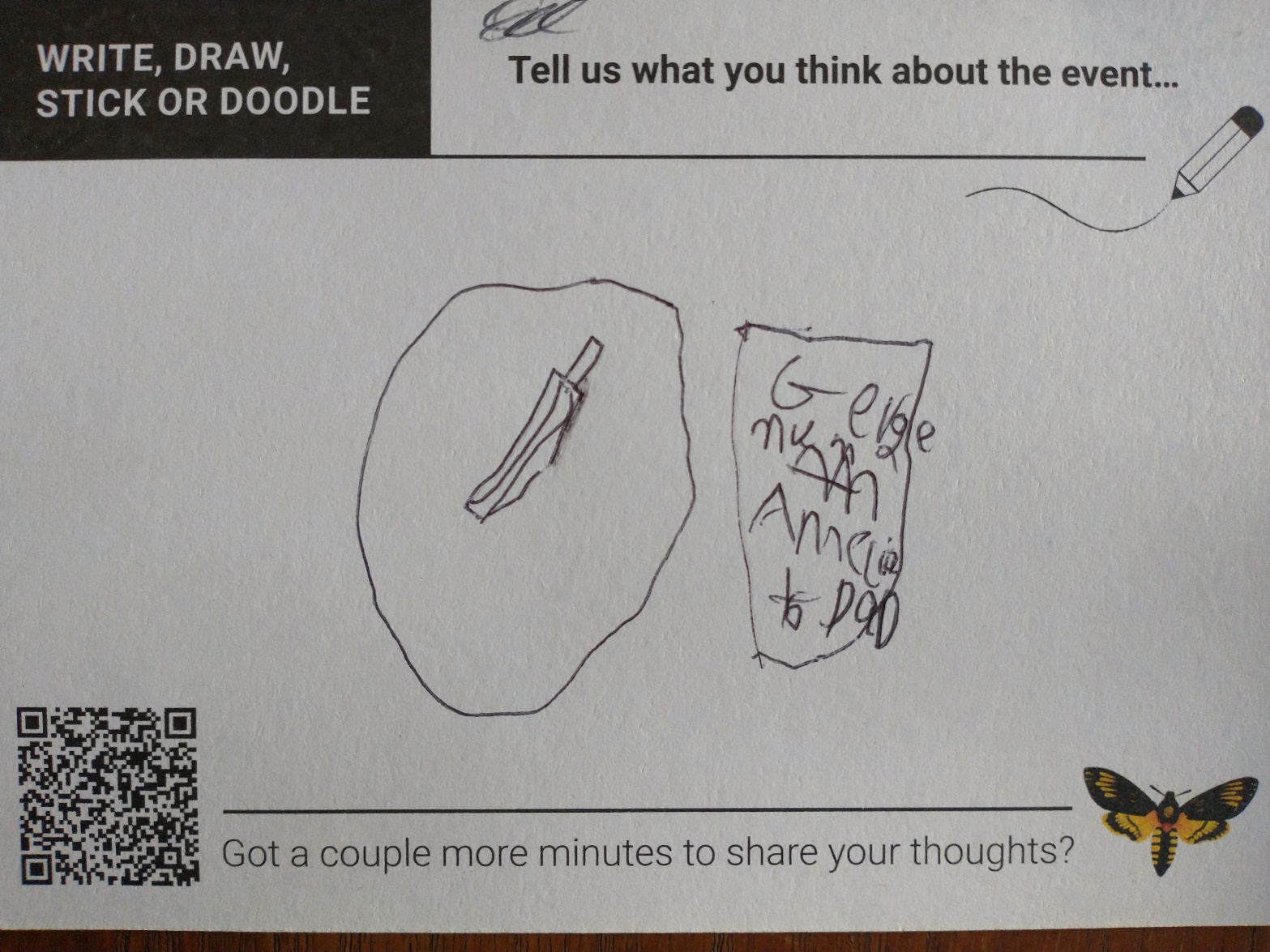


PC15


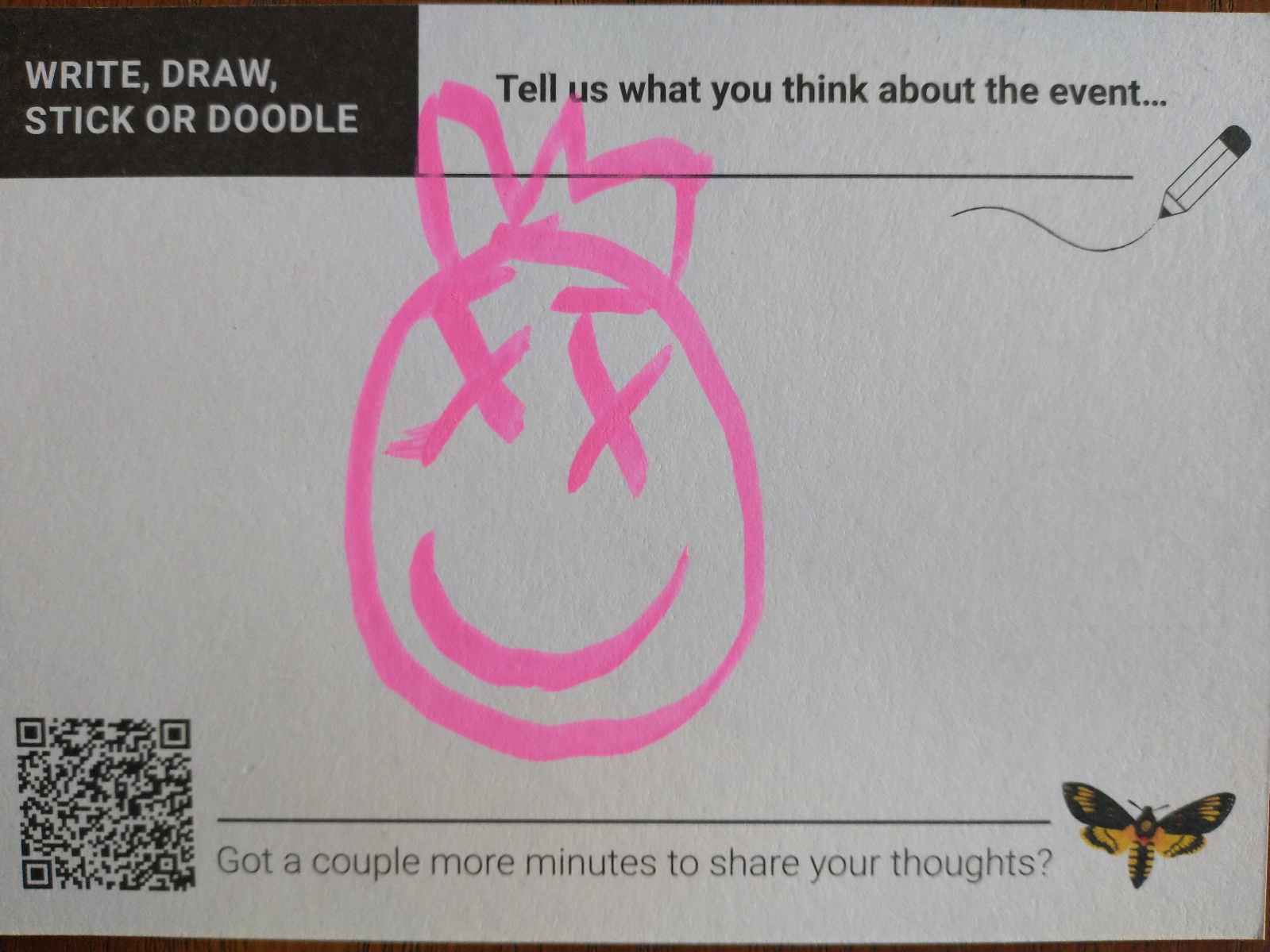


PC20


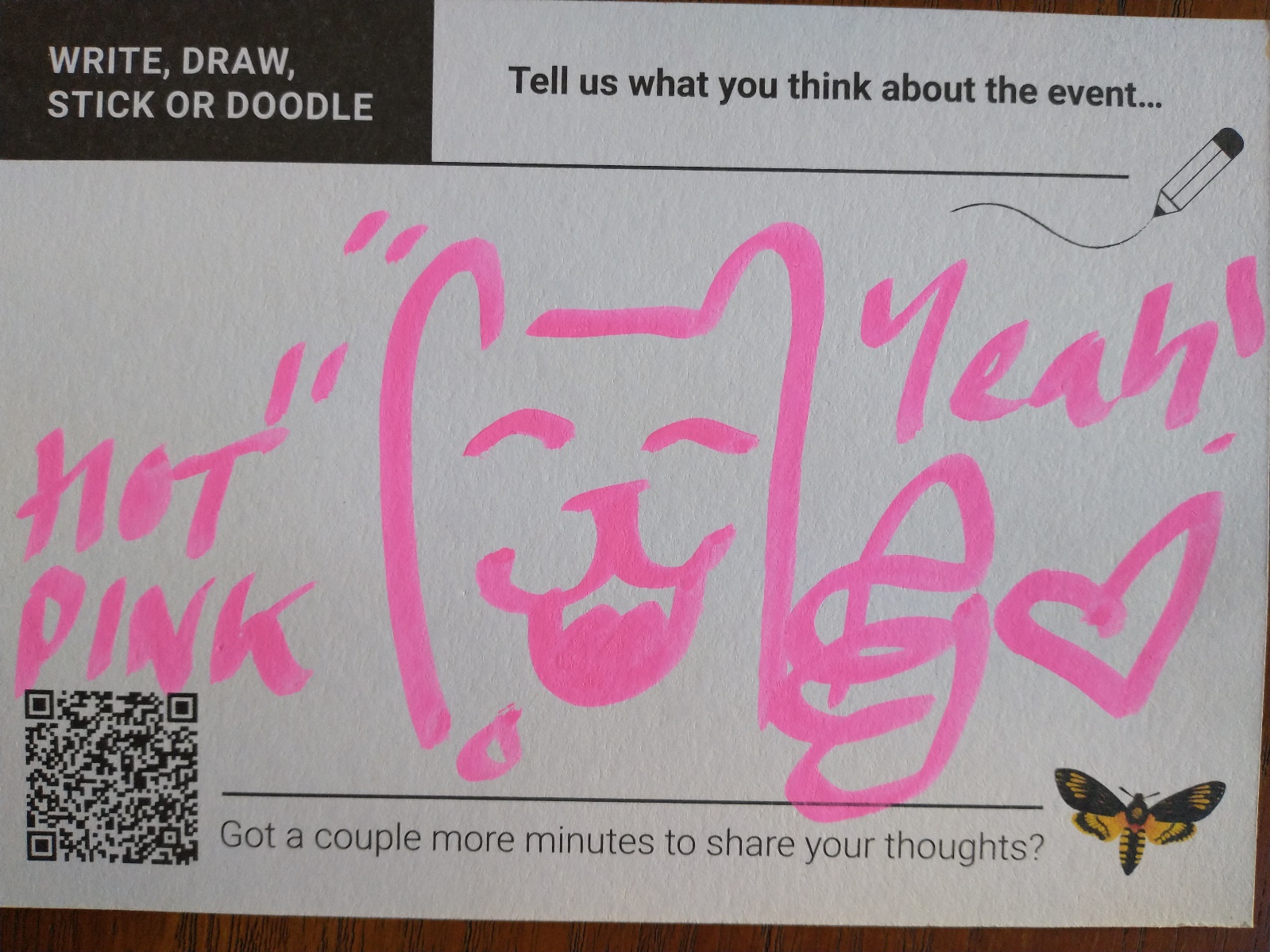


PC21


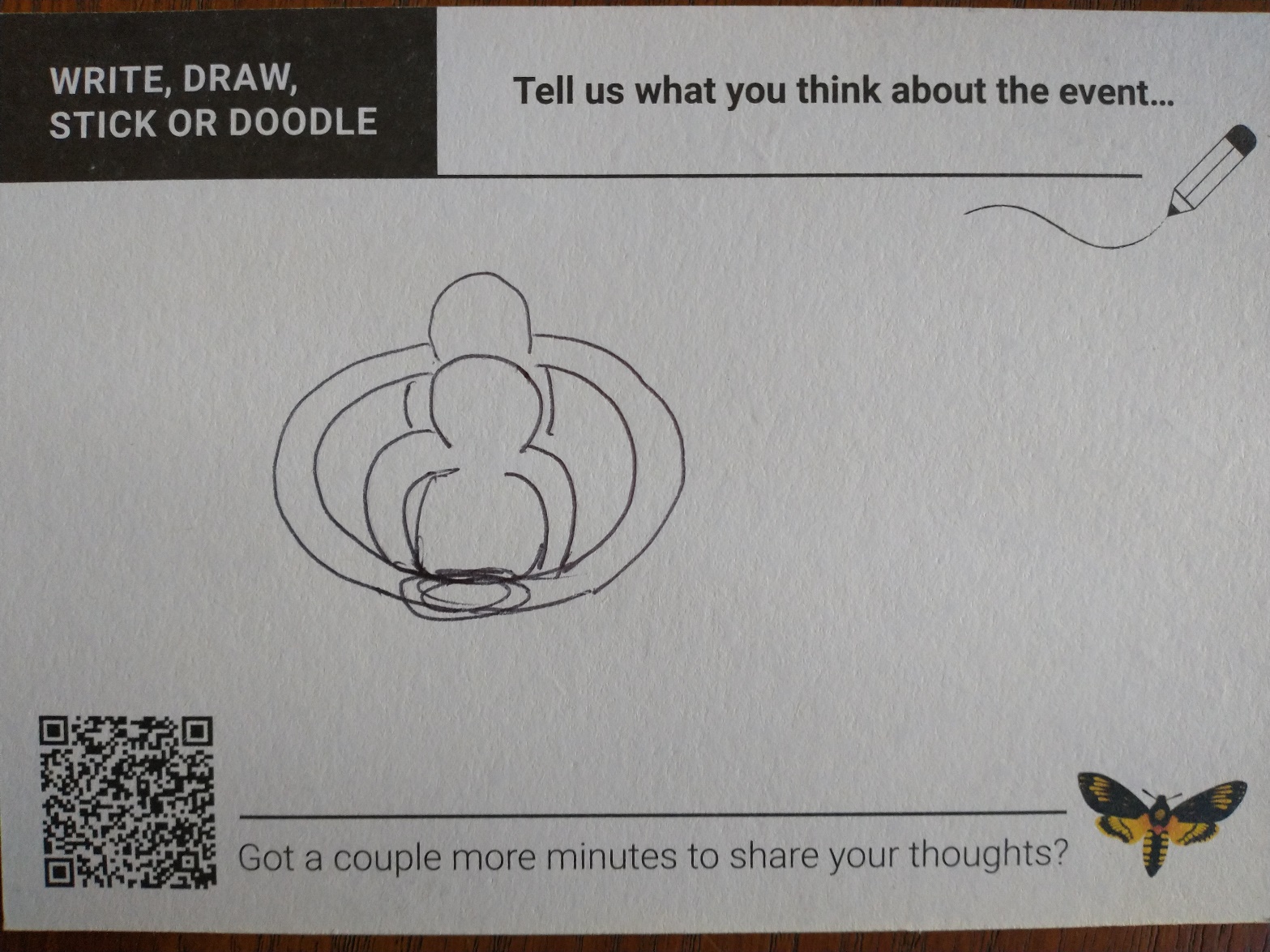


PC27


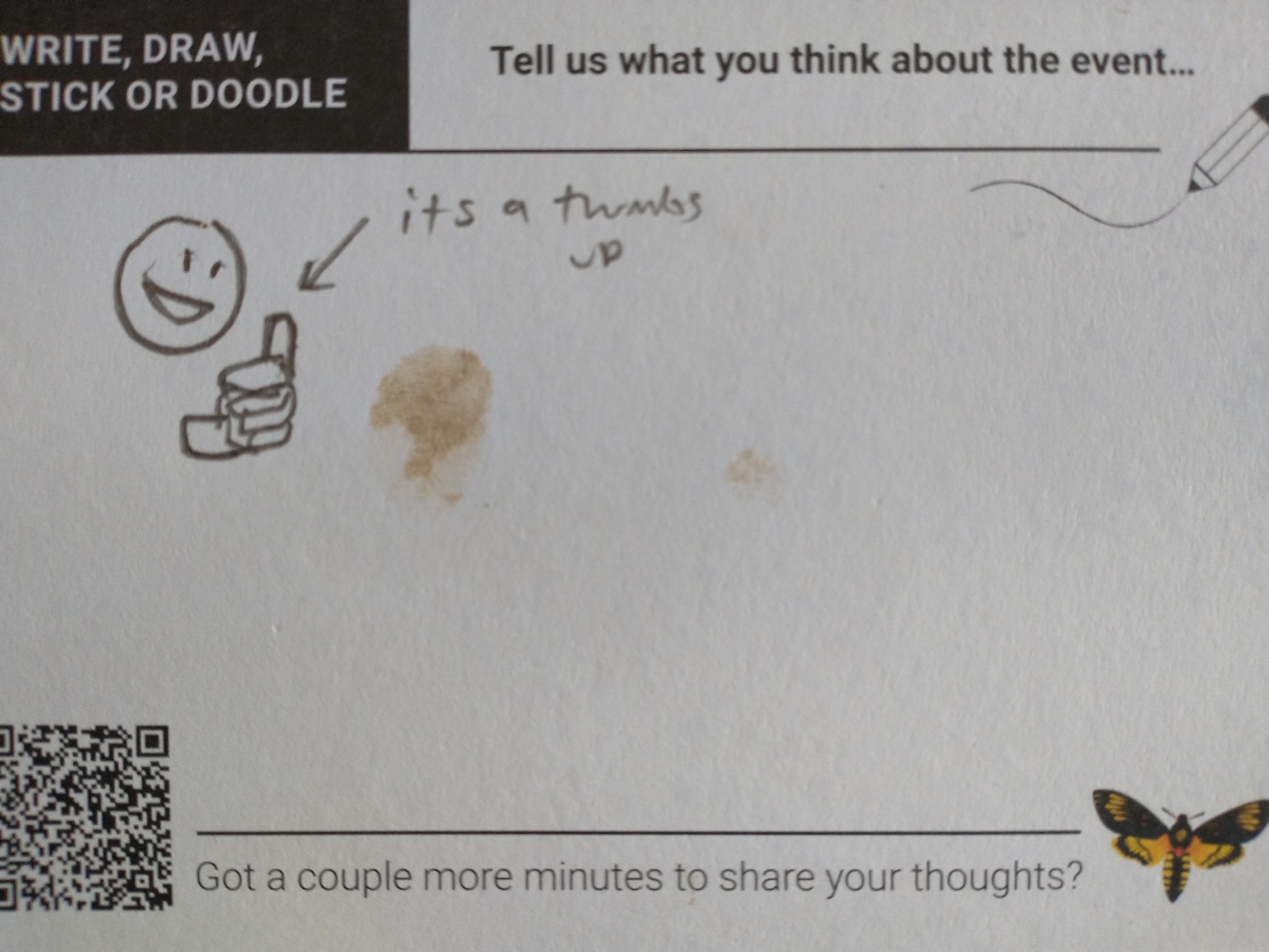


PC41


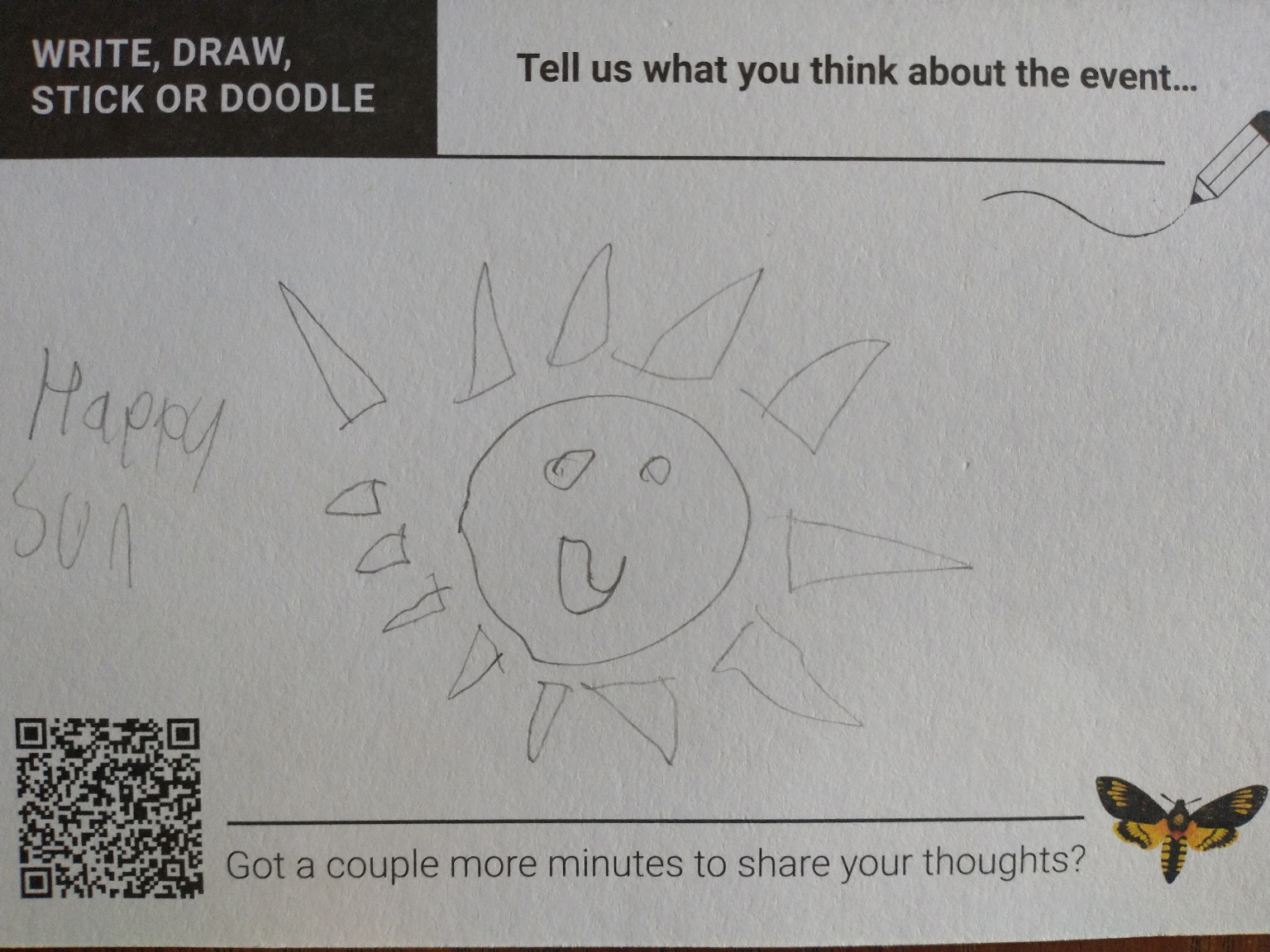


PC43


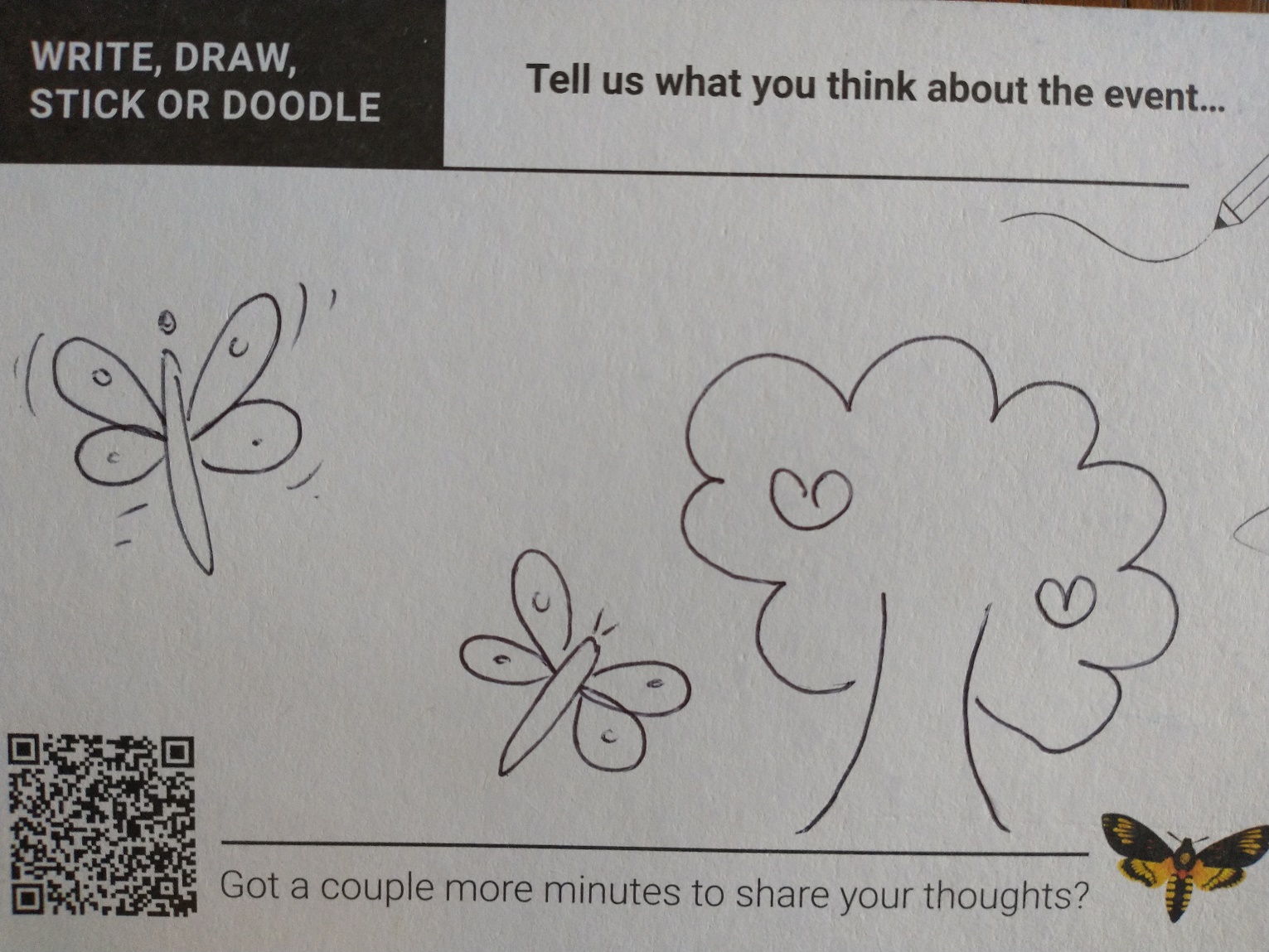


PC44


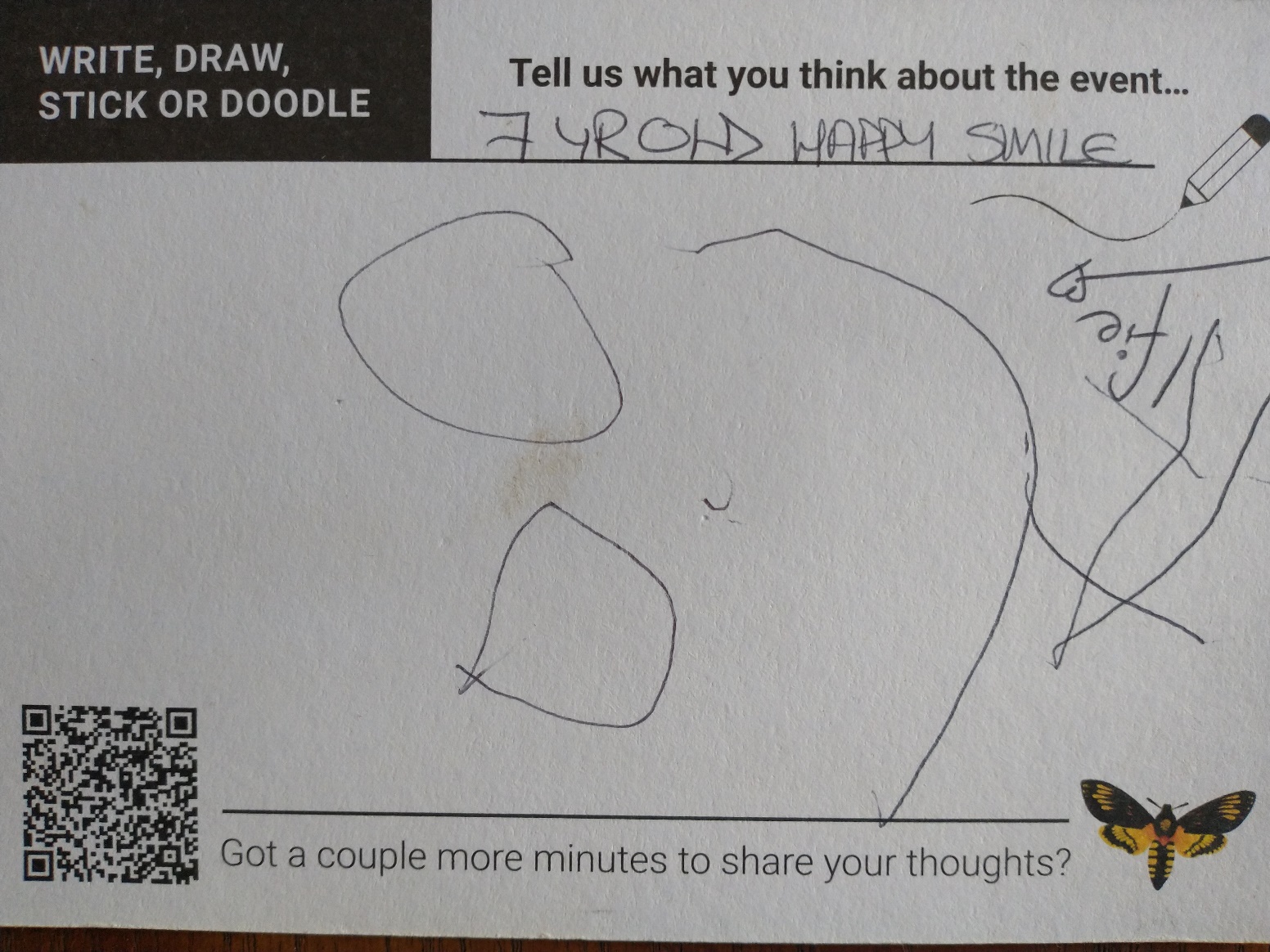


PC50

Supplement: sj-docx-6-pcr-10.1177_26323524241274175 – Supplemental material for Community engagement in a seaside town: evaluation of Good Grief Weston festival [file sj-docx-6-pcr-10.1177_26323524241274175.docx]

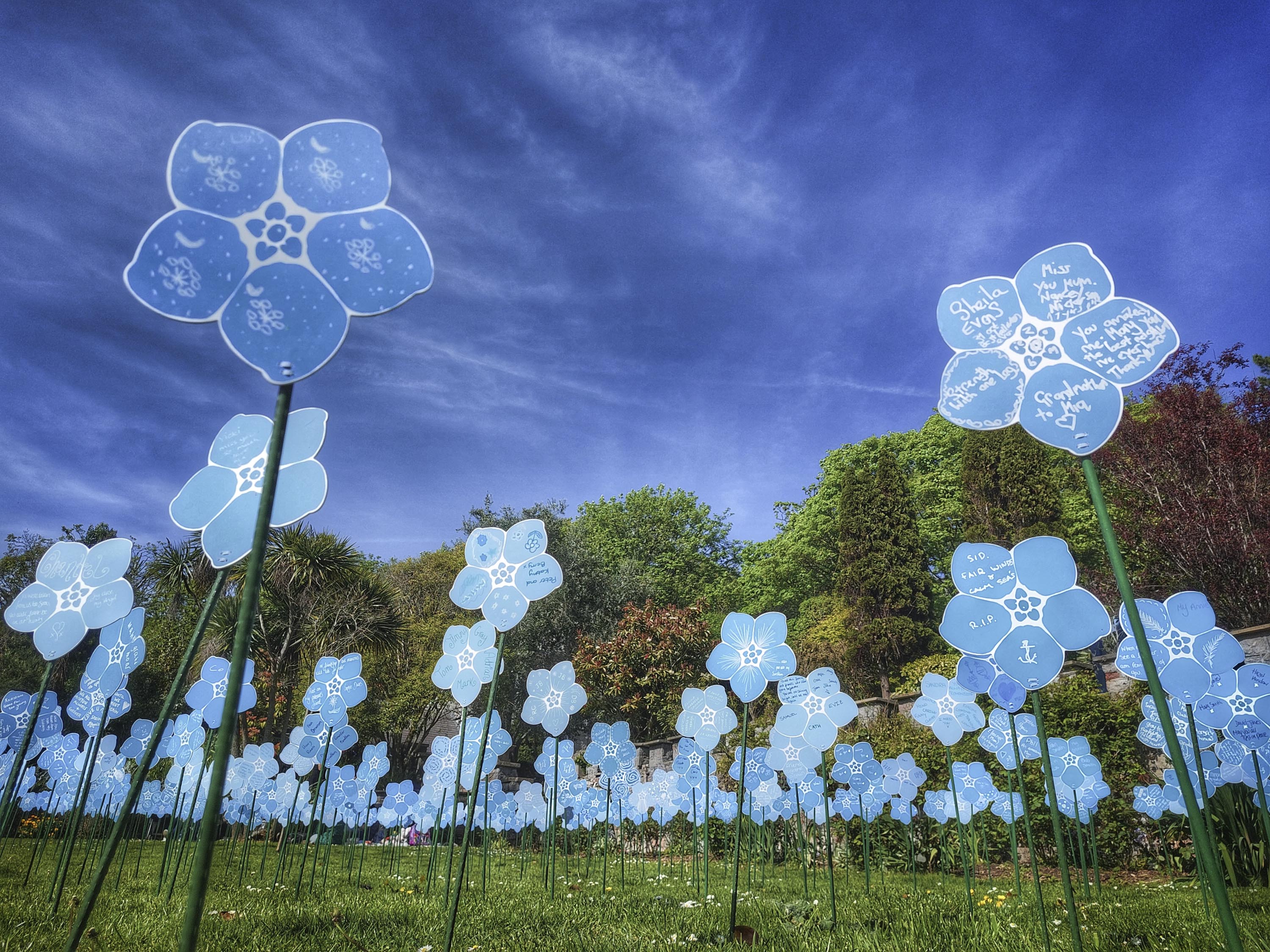

Supplement: sj-jpg-2-pcr-10.1177_26323524241274175 – Supplemental material for Community engagement in a seaside town: evaluation of Good Grief Weston festival [file sj-jpg-2-pcr-10.1177_26323524241274175.jpg]
