# Supplementary material for: Community engagement in a seaside town: evaluation of Good Grief Weston festival
Source: Palliat Care Soc Pract. 2024 Sep 6;18:26323524241274175. doi: 10.1177/26323524241274175 (PMC11378171; doi:10.1177/26323524241274175)
Supplement: sj-docx-3-pcr-10.1177_26323524241274175 – Supplemental material for Community engagement in a seaside town: evaluation of Good Grief Weston festival [file sj-docx-3-pcr-10.1177_26323524241274175.docx]

## Appendix 2: Post-festival survey (V2, 04.04.2023)

[Accessed by: i) email link to online survey sent to registered attendees; ii) QR code link on feedback cards/poster; iii) paper/hard copy printed out to complete in person]

**Survey text [adapted for posters/hard copy surveys]**

Thank you for completing this short survey.

The information you provide will help us understand the reach and impact of the festival. Responses are anonymous. Please complete the survey only once, even if you attended multiple events.

*By participating in this survey you agree that your responses may be used in future reports about the festival. All data collected in this survey will be held securely at the University of Bristol in accordance with the General Data Protection Regulations (GDPR; EU 2016/679). For more information, please contact* [*lucy.selman@bristol.ac.uk*](mailto:lucy.selman@bristol.ac.uk)

1. **How many festival events or activities did you attend?**

- 1 or 2
- 3-5
- More than 5
- I did not attend in the end (please tell us why) [open text response]:

1. Which activity or events did you attend?

[all events in the programme listed – plus ‘other’ – please state option]

1. **At the event(s) I** (*tick all that apply):*

- Was inspired
- Was entertained
- Talked to someone new
- Felt part of a like-minded community
- Shared or expressed my experiences
- Learnt about grief and bereavement
- Found out about local bereavement support or other support services e.g. community groups
- Other (please specify): [open text response]

1. **To what extent do you agree with the following statements*:*** *through attending the festival I feel more confident talking about grief.*

- Strongly agree
- Tend to agree
- Neither agree nor disagree
- Tend to disagree
- Strongly disagree
- Don’t know
- I already felt as confident as I could

1. **How would you rate your experience overall?** *(1=poor to 5=excellent)*

[slide/likert scale]

1. **Is there anything else you would like to say about Good Grief Weston?**

[open text response]

1. **Please tell us your suggestions for how the festival could be improved next year**

[open text response]

1. **To what extent do you agree or disagree, if at all, with the following statements about talking to someone who has recently experienced the death of a relative, partner or close friend.**
2. I would be scared of ‘saying the wrong thing’ to someone who was recently bereaved

- Strongly agree
- Tend to agree
- Tend to disagree
- Strongly disagree
- Don’t know

1. I would avoid talking to someone who was recently bereaved about their bereavement because I wouldn’t know how to help

- Strongly agree
- Tend to agree
- Tend to disagree
- Strongly disagree
- Don’t know

1. I would know what to do if someone who was recently bereaved told me they were having trouble

- Strongly agree
- Tend to agree
- Tend to disagree
- Strongly disagree
- Don’t know

1. I would know what kind of help or support to offer someone who was bereaved

- Strongly agree
- Tend to agree
- Tend to disagree
- Strongly disagree
- Don’t know

1. **If you experienced a bereavement or were supporting someone through serious illness or bereavement, where would you get local support?**
   - GP/doctors
   - Weston Hospicecare
   - Human Resources at work
   - School/college/university
   - Church/faith organisation
   - Community group (please state below)
   - Other (please state below)
   - I wouldn’t look for support, I would rely on family and friends
   - [open text response]
2. **To what extent do you agree with the following statement:** *Through attending the festival I have learnt about local services and support available for people living with serious illness or bereavement.*

- Strongly agree
- Tend to agree
- Neither agree nor disagree
- Tend to disagree
- Strongly disagree
- Don’t know

[Demographic questions required from Culture Weston (Q11-Q27, inclusive]

1. **What is your age?**

0-15* (go straight to Q24)

16-19

20-24

25-29

30-34

35-39

40-44

45-49

50-54

55-59

60-64

65-69

70-74

75+

Prefer not to say

1. **What is your sex?**

Female

Intersex

Male

Prefer not to say

1. **Is your gender identity the same as the sex you were assigned at birth?**

No

Yes

Prefer not to say

1. **How would you describe your gender identity?**

Man

Non-binary

Woman

Prefer to self-describe [free text]

Not applicable

Prefer not to say

1. **With which ethnicity do you identify?**

Arab

Asian/Asian British: Bangladeshi

Asian/Asian British: Chinese

Asian/Asian British: Indian

Asian/Asian British: Pakistani

Black/Black British: Black African

Black/Black British: Black Caribbean

Latin American

Mixed background: Asian & white

Mixed background: Black African & white

Mixed background: Black Caribbean & white

White/White British: Gypsy, Roma or Irish traveller

White/White British: White British

White/White British: White Irish

Prefer to self-describe [free text]

Prefer not to say

Not known

1. **Do you identify as a D/deaf and/or D/disabled person, or have a long term health condition?**

Yes

No

Prefer not to say

1. **Do you identify as neurodivergent? Being Neurodivergent could include Dyslexia, ADHD etc.**

Yes

No

Prefer not to say

1. **What best describes your current or most recent occupation?**

- **Clerical and intermediate occupations** such as: secretary, personal assistant, call centre agent, clerical worker, nursery nurse.
- **Full-time education** such as: studying for a degree or apprenticeship
- **Long term unemployed** (claimed Jobseeker’s Allowance or earlier unemployment benefit for more than a year).
- **Modern professional & traditional professional occupations** such as: teacher, nurse, physiotherapist, social worker, musician, police officer (sergeant or above), software designer, accountant, solicitor, medical practitioner, scientist, civil / mechanical engineer.
- **Retired**
- **Routine, semi-routine manual and service occupations** such as: postal worker, machine operative, security guard, caretaker, farm worker, catering assistant, sales assistant, HGV driver, cleaner, porter, packer, labourer, waiter/waitress, bar staff.
- **Senior, middle or junior managers or administrators** such as: finance manager, chief executive, large business owner, office manager, retail manager, bank manager, restaurant manager, warehouse manager.
- **Small business owners** who employed less than 25 people such as: corner shop owners, small plumbing companies, retail shop owner, single restaurant or cafe owner, taxi owner, garage owner.
- **Technical and craft occupations** such as: motor mechanic, plumber, printer, electrician, gardener, train driver.
- **Not applicable** (eg not currently working and not claiming benefits related to unemployment)
- **Prefer not to say**
- **Not known**
- **Other – please specify**

1. **If you would like to expand on the above, please tell us what your current or most recent occupation is in your own words**

[open text response]

1. **How would you describe your sexual orientation?**

Bisexual

Gay Man

Gay Woman / Lesbian

Heterosexual / Straight

Queer

Prefer to self-describe [free text]

Prefer not to say

Not known

1. **Do you currently reside in the UK?**

Yes

No

1. **If Yes: what is the postcode of your current residence?**

[open text response]

1. **If No: what is your country of residence (if not UK)**

[open text response]

1. **Who did you attend with?**

By myself

Friends and/or family

School/College/University visit

Organised group visit (not with school/college/university)

Other - please specify [open text response]

1. **With how many people of each age group did you attend (excluding yourself)?**

Age 0-4 [insert number]

Age 5-11 [insert number]

Age 12-15 [insert number]

Age 16+ [insert number]

1. **When did you last attend a Culture Weston event?**

0 < 6 months

6 < 12 months

Last 1 < 2 years

Last 2 < 3 years

More than 3 years ago

I have never attended before

1. **How often do you attend or participate in arts and culture experiences?** By this we mean online or in-person activities produced by arts organisations, museums and/or libraries, such as exhibitions, performances, literature events, gigs, festivals, creative participation etc.

This is my first time

Less than once a year

Once a year

2-3 times a year

4-5 times a year

6+ a year

1. **Finally, would you be willing to take part in a focus group about the festival?** If you would like to find more, please enter your contact details below (telephone and/or email address – as preferred). It will not be shared or used for any other purpose. Focus group participants will be paid £25 as a thank you for participating.

[open text response]

**That’s all of the questions. Please click ‘submit’ when you’re ready.**

**Submit**

Thank you for completing this survey

The information you have provided will help us understand the reach and impact of the festival.

If you have any further questions, please contact us [email]
