# Supplementary material for: Community engagement in a seaside town: evaluation of Good Grief Weston festival
Source: Palliat Care Soc Pract. 2024 Sep 6;18:26323524241274175. doi: 10.1177/26323524241274175 (PMC11378171; doi:10.1177/26323524241274175)
Supplement: sj-docx-5-pcr-10.1177_26323524241274175 – Supplemental material for Community engagement in a seaside town: evaluation of Good Grief Weston festival [file sj-docx-5-pcr-10.1177_26323524241274175.docx]

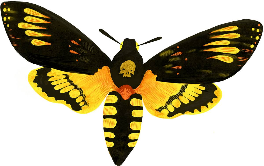

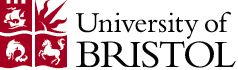

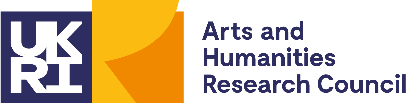


## Supplementary file 5: After festival focus group topic guide

**Introduction**

- Explanation of study aims and audio recording permission
- Audio consent to be taken/recorded
- Names/introductions

**Festival**

1. What made you want to attend the festival?
   - Prompt: how did you hear about it?
2. What were your overall impressions of the festival before the event? Probe e.g.

- how it was advertised/marketed (Did it seem to be directed at you? How inclusive did it appear to be?)
- registration process
- views of communication before the event

1. What were you hoping for from the festival?
2. Did you attend alone or with others?
   - If so, who and why?
3. What did you think of the festival overall?
4. What did you attend?
5. What did you find most useful?
6. Did you learn anything new or not?
   - Explore which topics
7. Was there anything missing?
   - Particular topics, activities, or artistic methods
8. How involved did you feel?
   - Was it interactive? Engaging?
9. Did you feel able to share your experiences where you wanted to?
   - If so, how? If not, why not?
10. Do you think the festival was welcoming to all? For example:
    - What about young people
    - Older people
    - People from diverse ethnic minority communities
11. Are there any people/groups who may have wanted to attend but have been unable to?
    - If so, who and why?
12. What did you think of the festival being in person (not online)?
13. Did you find the festival was pitched correctly?
    - Was it understandable/accessible?
14. What didn’t work for you?
    - Any insensitivities? Did you feel there was enough support/signposting to support? Level of participation required/possible?
15. What do you think we should do differently next time?
16. Are there any local community groups or organisations that you have discovered through your participation in the festival?

**Thanks and close**

1. Is there anything else you’d like add?
   - Turn off audio recorder. Thank participants and ensure they have completed the Participant Details form and indicated if they want to receive a copy of the summary report.
